# Impact of augmented reality on narrative-related outcomes in children with Autism Spectrum Disorder: a within-subject design

**DOI:** 10.3389/fpsyg.2026.1926566

**Published:** 2026-09-25

**Authors:** Carmen Berenguer, Cristina Fuentes-Sáez, Inmaculada Baixauli, Ma de El Puig Andrés, Soledad Gómez-García

**Affiliations:** 1 Department of Developmental and Educational Psychology, ERI-Lectura, Faculty of Psychology and Speech Therapy, University of Valencia, Valencia, Spain; 2 Doctoral School, Catholic University of Valencia San Vicente Mártir, Valencia, Spain; 3 Catholic University of Valencia San Vicente Mártir, Valencia, Spain

**Keywords:** assistive technology, augmented reality, Autism Spectrum Disorder, engagement, narrative, Theory of Mind

## Abstract

**Inroduction:**

Autism Spectrum Disorder (ASD) is commonly associated with impairments in social communication, narrative abilities, and socio-cognitive functioning. This study explored whether augmented reality (AR) could enhance narrative production, comprehension, use of mental state language, and reading-related behaviors in children with ASD.

**Methods:**

Fifteen children with ASD without intellectual disability participated in a within-subjects design in which they completed story retelling tasks under two counterbalanced conditions: a traditional paper-based format and a tablet-based format. Narrative performance was assessed through measures of microstructure, including productivity, lexical diversity, and syntax, as well as macrostructure indicators such as recall of main ideas. Oral comprehension and reading-oriented behaviors were also examined.

**Results:**

Results showed that the AR condition significantly improved socio-cognitive and behavioral outcomes. Children produced a higher number of mental state terms and demonstrated greater attention, persistence, and engagement during the tasks. These findings suggest that AR can function as an effective scaffold that facilitates the explicit representation of internal states while increasing attentional involvement.

**Discussion:**

Consequently, AR technology may represent a promising tool for improving language assessment and fostering social-cognitive development in children with ASD.

## 1 Introduction

Autism Spectrum Disorder (ASD) is a neurodevelopmental condition characterized by persistent difficulties in social communication and interaction, alongside restricted and repetitive patterns of behavior, interests, or activities (American Psychological Association, 2020). Narrative discourse is a fundamental pragmatic competence, as it integrates linguistic, cognitive, and social abilities, allowing individuals to organize events, consider the listener’s perspective, and communicate experiences coherently (Norbury et al., 2014). Given its inherently social and cognitively demanding nature, this type of discourse provides a particularly valuable context for both assessment and intervention in ASD (Hewitt, 2019).

Narrative abilities in neurodevelopmental conditions are commonly examined across two complementary levels: microstructure and macrostructure. Microstructure refers to local linguistic features such as productivity, lexical diversity, and syntactic complexity, whereas macrostructure concerns global aspects of organization, coherence, and thematic development (Hughes et al., 1997). In ASD, findings regarding microstructural performance are mixed. A meta-analysis by Baixauli et al. (2016) reported that individuals with ASD without intellectual disability tend to perform poorer than typically developing peers in measures such as total word production, utterance length, lexical diversity, and grammatical complexity, with moderate effect sizes. However, results across studies remain heterogeneous: some report comparable performance in narrative length or syntactic complexity (Diehl et al., 2006; Norbury and Bishop, 2003), whereas others describe reduced lexical variety, shorter narratives, and increased linguistic errors (Adornetti et al., 2024; Peristeri et al., 2017). In contrast, macrostructural impairments appear more robust and consistent across studies and languages, with large effect sizes (Baixauli et al., 2016). Individuals with ASD frequently show difficulties in maintaining global coherence, organizing events causally, and constructing well-structured narrative episodes (Adornetti et al., 2024; Ferretti et al., 2018). They also tend to produce fewer references to internal states (e.g., thoughts, emotions, intentions) (Losh and Capps, 2003; Peristeri et al., 2017), which are closely linked to Theory of Mind, that is, the ability to attribute mental states to oneself and to others, making it possible to explain and predict behavior (Premack and Woodruff, 1978). Importantly, cross-linguistic evidence suggests that macrostructural difficulties persist even when microstructural performance is relatively preserved, particularly in coherence, relevance selection, and episodic organization (Adornetti et al., 2024; Domingo et al., 2026; He et al., 2025; Peristeri et al., 2025).

### 1.1 Impact of multimedia features on narrative assessment

A key issue in narrative assessment is the variability in performance as a function of the elicitation task and administration procedures. A wide range of methods is used, including fictional story generation, personal narratives, and story retelling, with or without visual support (Kazemi et al., 2014). Because these tasks impose different linguistic and cognitive demands, storytelling and retelling often yield distinct production outcomes and provide complementary insights into linguistic and conceptual abilities (Duinmeijer et al., 2012; Gutiérrez-Clellen et al., 2000).

Furthermore, elicitation materials such as paper picture books, digital picture books, audio, and video have also been shown to differentially influence children’s narrative production. In early development, visual aids play a central role, with visually supported narratives generally showing better grammatical organization than those elicited through auditory input alone (Schneider and Dubé, 1997). Additionally, beyond the presence of visual input, the format of presentation appears to be relevant. In recent years, increasing attention has been given to the role of multimedia features and digital technologies in language assessment and intervention, including video, animation, audio, music, and interactive tools (e.g., tablets and software applications). In typically developing populations, findings about the advantages of these multimedia features in narrative assessment remain mixed. Gazella and Stockman (2003) found no differences between audio and audiovisual conditions in microstructural measures such as the amount of speech, lexical diversity, or syntactic complexity. Likewise, Klop and Engelbrecht (2013) reported no significant differences between silent video and wordless picture-book conditions in narrative complexity, although the limited sample size may have constrained statistical power. In contrast, Diehm et al. (2020) found that video-supported retelling elicited longer narratives, with greater lexical diversity and more syntactically complex structures, as well as a reduced need for prompts, suggesting improved fluency and clearer access to dynamic event information.

Another way to elicit narrative language in children is through diverse story formats, including traditional print books, e-books, interactive books, and multimedia story applications. The increasing availability of both print and digital formats provides clinicians with a range of tools that offer distinct possibilities for eliciting narrative language (e.g., Kucirkova, 2019). While print books rely on static illustrations accompanying written text, digital formats incorporate features such as animation, sound and narration, which can enrich the storytelling experience and promote engagement (O’Byrne et al., 2018). However, evidence suggests that, although electronic storybooks tend to increase children’s attention, engagement, and persistence, they do not necessarily lead to improved story recall when compared to traditional print formats (Richter and Courage, 2017). Similarly, research by Lauricella et al. (2014) examining parent–child interactions during shared reading found that, although parental engagement was higher with computer-based storybooks, children’s story comprehension did not significantly differ between traditional and digital formats.

### 1.2 Impact of augmented reality on narrative assessment

More recently, augmented reality (AR) has emerged as an innovative approach that integrates physical and digital elements to create immersive storytelling experiences. AR technology overlays virtual content—such as text, audio, video, and animated characters—onto real-world environments, enabling interactive and multimodal engagement with story content (Leung and Blauw, 2020). In the context of storybooks, AR has been shown to promote active participation, enhance motivation, and support deeper narrative engagement through interactive features such as virtual pop-ups and touch-based exploration (Kljun et al., 2020).

There is initial evidence that supports the potential of AR storybooks to enhance narrative outcomes. For instance, a recent study found that typically developing preschool children exposed to AR-enhanced storybooks demonstrated significantly better story retelling and comprehension compared to peers who experienced the same stories in print format (Şimşek, 2024). These findings suggest that AR may provide added value not only in terms of engagement but also in supporting narrative production, a key component of language elicitation in assessment contexts. Regarding the population with ASD, given the well-documented preference of these children for visual stimuli and structured input, AR may represent a particularly suitable tool for supporting their language and narrative abilities. In fact, AR technologies have shown a positive effect on improving different domains such as social communication skills, verbal and nonverbal communication, facial emotion recognition (Berenguer et al., 2020) and learning outcomes in individuals with this condition (Fuentes et al., 2025).

In the specific domain of narrative comprehension and production, AR may be particularly beneficial. By enhancing salient visual cues within stories, AR can facilitate attentional focus and sustained engagement (Escobedo et al., 2014), as well as increase motivation for learning. It may also help mitigate difficulties related to weak central coherence (Happé and Frith, 2006), a cognitive style commonly associated with autism and characterized by a tendency to focus on local details rather than integrating information into a coherent whole. Therefore, deficits in central coherence might be addressed through the use of AR by guiding attention toward relevant elements of the narrative, such as emotional expressions, causal relationships, and key events. The preliminary research on the use of AR to enhance language abilities in individuals with ASD provides promising insights. For instance, Chen et al. (2016) examined the use of AR combined with video modeling to improve the processing of nonverbal facial cues within narrative contexts for adolescents with high-functioning ASD. While participants initially focused on irrelevant details and struggled to interpret emotional and social cues, the intervention helped them shift their attention toward relevant information, resulting in an improved understanding of characters’ emotions and narrative meaning. In addition, Wainwright et al. (2020) investigated the effects of presentation media (print vs. e-book) and narration types (adult vs. app-based) on narrative comprehension and engagement in children with ASD, in comparison to typically developing peers. Although e-books attracted greater visual attention in both groups, this condition did not translate into improved comprehension for children with ASD. In contrast, a positive association between visual attention and comprehension was observed in typically developing children. Moreover, print-based reading proved more time-efficient, suggesting that while digital formats may enhance attentional engagement, traditional books remain equally effective at supporting narrative comprehension in children with ASD.

### 1.3 Objectives

The primary aim of this study was to examine the differential effects of the AR narrative elicitation mode in children with ASD, compared to the traditional paper elicitation mode, on narrative production and comprehension, socio-cognitive indicators, and behaviors oriented toward reading. Specifically, the study sought to determine whether the AR condition would lead to greater improvements in these domains. The study was structured around the following objectives and hypotheses:

The first objective was to evaluate the impact on reading-oriented behaviors. It was hypothesized that the AR condition would result in higher levels of reading-oriented behaviors, including increased sustained attention and engagement, and reduced distractibility. This is based on findings suggesting that digital formats can enhance children’s involvement through interactive presentation (Richter and Courage, 2017).

The second objective was to examine the effect of AR on narrative production (microstructure and macrostructure) and comprehension. Based on the premise that dynamic visual support facilitates access to information regarding actions (Diehm et al., 2020), it was expected that children in the AR condition would show advantages in both narrative dimensions compared to those in the paper condition. Moreover, regarding comprehension, it was hypothesized that the multimodal input provided by AR would facilitate the interpretation of narrative events, leading to higher comprehension scores by offering a more explicit representation of the story’s causal sequence.

Finally, the third objective was to analyze the influence of AR on the use of socio-cognitive indicators such as mental state terms. It was hypothesized that the AR condition would promote greater use of these types of terms. The dynamic nature of AR was expected to act as a scaffold for interpreting characters’ internal states, which are often difficult for children with ASD to decode in static formats (Wainwright et al., 2020).

## 2 Methods

### 2.1 Participants

Fifteen children with Autism Spectrum Disorder (ASD) were recruited from three educational and clinical settings located in Valencia, Spain. Specifically, the final sample comprised 12 males and 3 females, with a mean age of 6 years and 6 months and a mean composite IQ of 90.2. Regarding medication status, only one participant had been receiving pharmacological treatment since January 2026, whereas none of the remaining children across the three centers was under medication at the time of assessment (Table 1). Participants were included according to the following criteria: (a) a composite IQ above 70, assessed using the Kaufman Brief Intelligence Test (K-BIT; Kaufman and Kaufman, 2004); and (b) language abilities corresponding to Phase 4 of the developmental classification proposed by Tager-Flusberg et al. (2009). At this stage, children are able to combine words into clausal structures or sentences and use emerging morphological markers such as plurals, prepositions, and verbal morphemes. In addition, their vocabulary repertoire is sufficiently broad to meet everyday communicative needs, allowing them to use language across a wide range of communicative functions and social contexts with both familiar and unfamiliar interlocutors.

**Table 1:** Sociodemographics.

ASD (*M* = 15)	*M*	SD
Age	6.61	0.88
IQ	90.23	7.73
Vocabulary	11.45	1.99
Parent education level	3.12	1.01
DSM-5 ASD	4.37	3.62
Sex (*n* % boys)	12	80%
Medication (*n* % yes)	1	6.6%

*M*, mean; SD, standard deviation; IQ, intelligence quotient; DSM-5 ASD, Autism Spectrum Disorder according to the Diagnostic and Statistical Manual of Mental Disorders, Fifth Edition; *n*, number of participants; Sex (*n* % boys), Sex (number and percentage of boys); Medication (*n* % yes), Number and percentage of participants taking medication. *p* < 0.05.

### 2.2 Materials and measures

The materials used in the assessment consisted of two stories with a simple structure in terms of both episodic organization and linguistic complexity, depicting everyday events in the life of a child, Ismael, and his family: “Ismael in the Countryside” and “Ismael at the Beach” (Gómez, 2019). Both narratives included references to emotional, intentional, and desire-related states of the characters. The two stories were carefully matched in terms of the number of episodes and illustrations. Specifically, both narratives comprised an introduction, five episodes, and a resolution, and were accompanied by thirteen illustrations. The plot structure and actions were also controlled to ensure equivalence across stimuli. In addition, the narrative materials were selected to guarantee comparability in both macrostructural and microstructural features, as well as cultural appropriateness. For a more detailed description of the materials, see Baixauli-Fortea et al. (2019).

Figure 1 illustrates a scene designed to elicit the description of mentalizing skills. The first image presents an example from the 2D book, which corresponds to the same scene later represented as a 3D illustration using augmented reality (AR). In Figure 2, a 2D scene is shown with the aim of emphasizing the emotion of sadness; correspondingly, the following image demonstrates how this scene appears in 3D when viewed through the AR application on a tablet.

**Figure 1:**
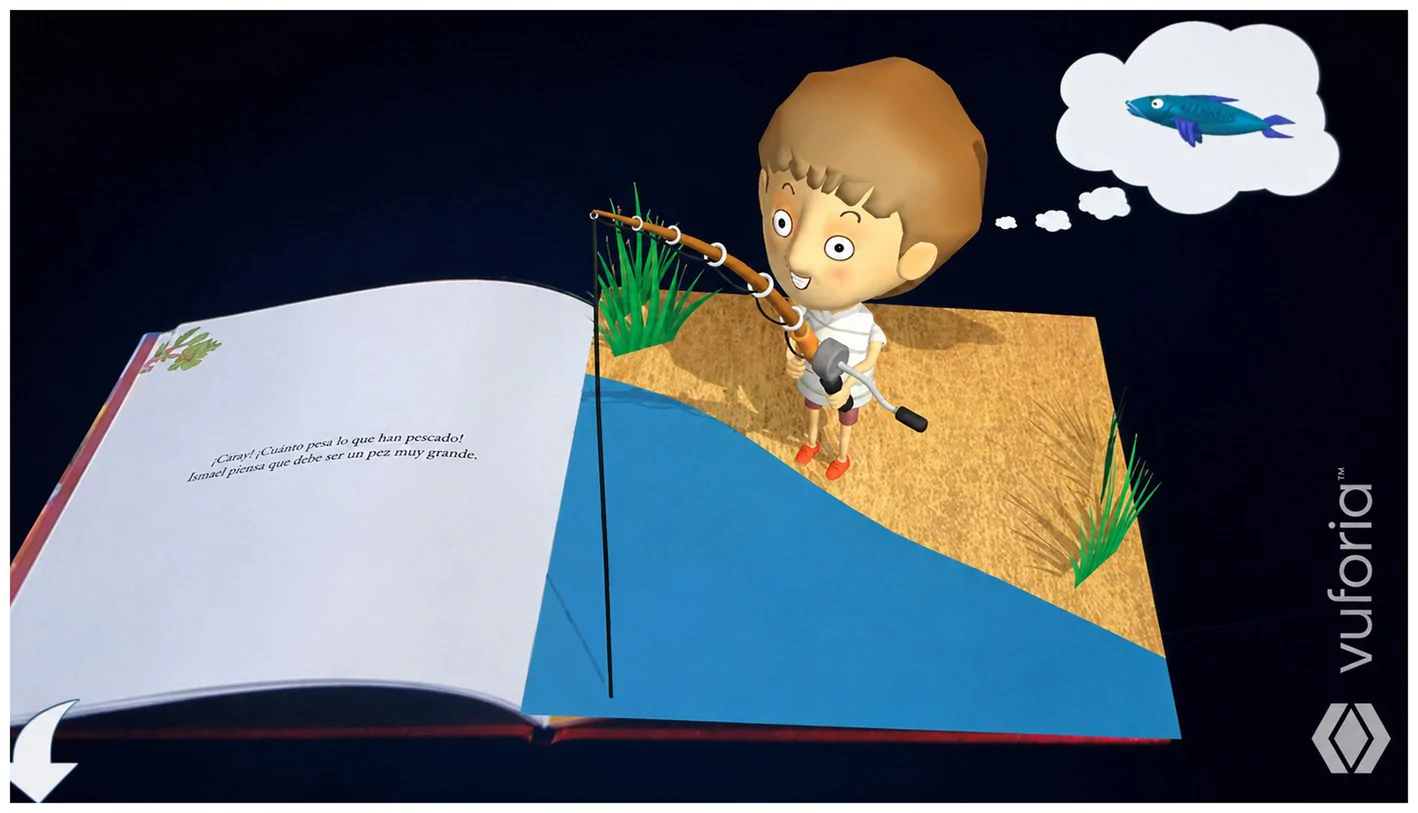
Ismael in the Countryside. Created by the authors.

**Figure 2:**
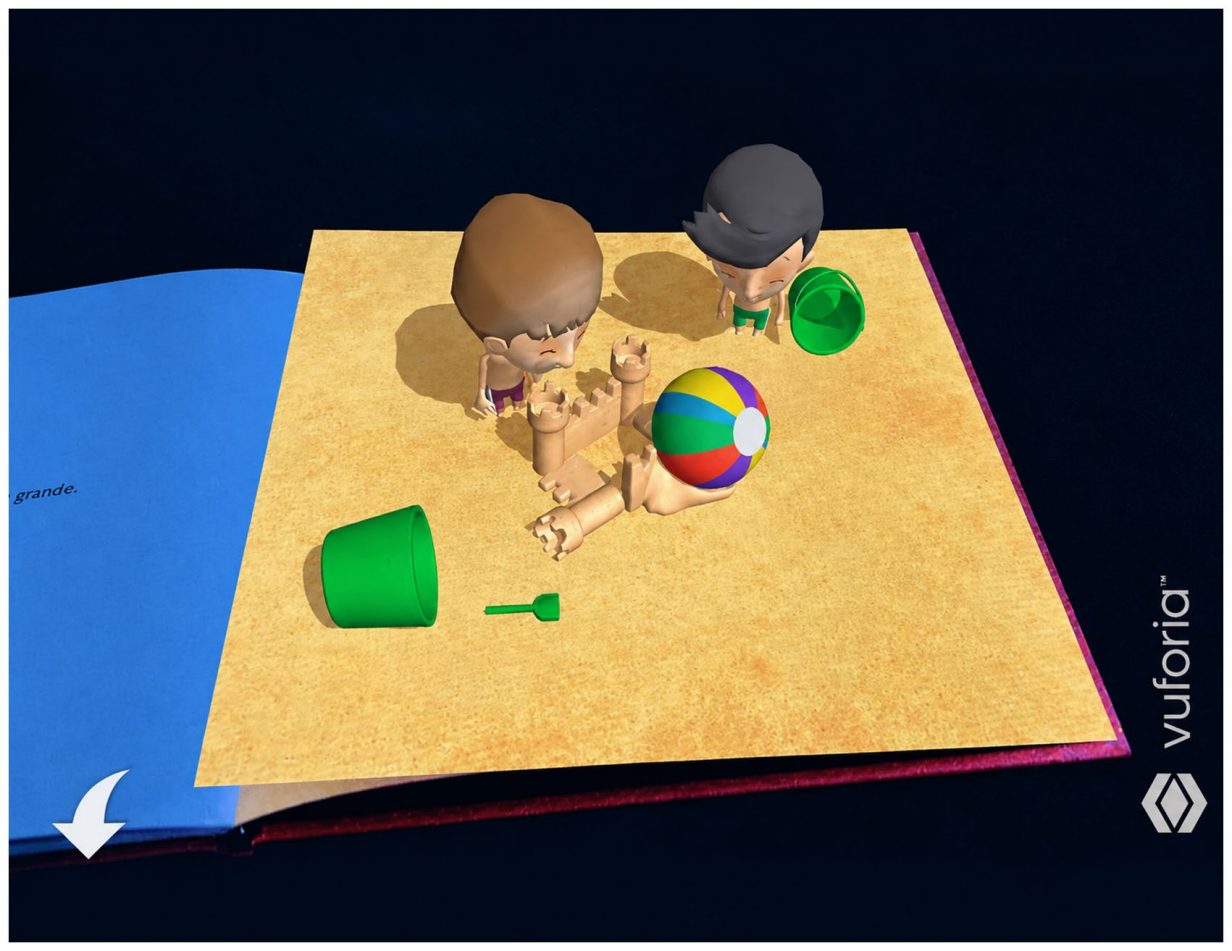
Ismael at the Beach. Created by the authors.

#### 2.2.1 Narrative production measures

The narratives were video-recorded, transcribed, segmented into utterances, and coded using the SALT program (Miller and Iglesias, 2012), in accordance with the variables described below.

##### 2.2.1.1 Microstructure measures

(i) Productivity, This was calculated as the number of utterances, based on the definition of an utterance as “an independent clause and any clauses dependent on it” (Hunt, 1965). This measure is automatically generated by the SALT software. (ii) Mean length of utterance (MLU): This was calculated by dividing the number of words by the number of utterances (Brown, 1973; Spanish adaptation by Aguado, 1995). This measure is automatically computed by SALT. (iii) Subordination index: As a measure of syntactic complexity, this was obtained by dividing the total number of subordinate clauses by the total number of utterances (Hunt, 1965). It is automatically computed by SALT. (iv) Lexical diversity: Two indicators were considered, both calculated by SALT: the number of different words and the type-token ratio (TTR), the latter obtained by dividing the number of different words by the total number of words (Richards, 1987).

##### 2.2.1.2 Macrostructure measures

(i) Main ideas: following the procedures described in narrative assessment tools (e.g., Bishop, 2004; Renfrew, 2010), the presence of key story events or main ideas was evaluated based on those extracted from each story text. Specifically, fully correct ideas produced by the child received 1 point. Converselly, half points (0.5) were awarded for: (a) partial information (e.g., in the story *Ismael Goes to the Beach*, the child mentions that the character is building a sandcastle but does not mention the involvement of another child who helps in the process); and (b) the use of vague, overly general terms, or words within the correct semantic field but lacking precision—for example, referring to the squirrel as an “animal” in the countryside story. (ii) Socio-cognitive indicators: mental state terms: Socio-cognitive indicators can be understood as linguistic markers that reflect the speaker’s ability to represent, attribute, and reason about internal mental states, both in themselves and in others, which is a core component of Theory of Mind and broader social cognition (Astington and Baird, 2005). Within this framework, mental state terms constitute a particularly relevant index because they explicitly encode perceptions, emotions, desires, and beliefs—dimensions that are fundamental for interpreting others’ intentions and understanding social interactions. (iii) Following the classification by O’Neill et al. (2004), mental state terms were operationalized as the number of references to internal states produced in each child’s narrative. These included perceptual states (e.g., see), emotional states (e.g., sad), desire states (e.g., want), and belief states (e.g., think, know). The use of such terms is considered a socio-cognitive indicator because it reflects the child’s ability to go beyond the purely descriptive level of events and instead incorporate representations of the protagonists’ internal experiences. In doing so, children demonstrate an emerging capacity to interpret behavior in terms of underlying mental causes, which is central to social understanding and narrative coherence.

#### 2.2.2 Narrative comprehension measures

Ten questions were developed for each story to evaluate both literal (e.g., “Who did Ismael go to the beach with?”) and inferential comprehension of the narratives (e.g., “What did Ismael think was in the water?”). Two points were awarded for a correct response that included all relevant details and was expressed in appropriate language.

One point was given for a response that was reasonable but omitted some relevant information. No points were awarded for incorrect or vague responses.

#### 2.2.3 Children’s orientation to book reading

The child’s orientation to book reading was assessed using the *Children’s Orientation to Book Reading (COB) Rating Scale* (Kaderavek et al., 2014). All sessions were video-recorded to allow for subsequent detailed behavioral coding using scale, an observational tool designed to capture children’s responses to literacy events by assessing levels of engagement, nonverbal and verbal behaviors, persistence, and attentional focus during book-based interactions. In the present study, the task was adapted to a clinical assessment context in which the speech and language therapist presented the story individually to each child, either in the printed condition or in the augmented reality condition, as appropriate. Although the original COB procedure was developed for naturalistic, whole-class shared reading activities implemented by teachers, in this study it was applied in a structured clinical setting, while maintaining the core observational dimensions of the scale.

To rate a child’s literacy orientation using the COB, reliable and trained observers—a speech-language therapist and a doctoral psychologist specialized in neurodevelopmental disorders who were not directly involved in the assessment sessions-, watched the children’s affective involvement over the entire reading session and then rated the children’s behavior on a 4-point scale with scores of 1 and 2 indicating overall low orientation and scores of 3 and 4 indicating high orientation. This training ensured familiarity with the coding system and the application of standardized scoring criteria. The variables considered in the COB are the following: (a) Overall impression: From a rating of 1 (The child is clearly not engaged during the book interaction) to a rating of 4 (The child is interested in the book reading throughout the reading session with minimal/no display of low orientation); (b) Persistence: From a rating of 1 (The child is engaged less than 30% of the book interaction) to a rating of 4 (The child is engaged at least 70% of the book interaction); (c) Posture: From a rating of 1 (A lack of forward-leaning posture; child may position himself or herself at the periphery of the group) to a rating of 4 (Consistent forward-leaning posture; child is likely to place himself or herself in a good position to participate in the book reading and may move closer to the teacher so he/she can see the book more clearly); (d) Eye gaze: From a rating of 1 (The child may look blankly around the room, looking at other children or preoccupied visually with a toy or other object. Child does not look at the book) to a rating of 4 (Eye gaze is focused on reader and text at least 70% of the story; child may be observed moving or adjusting posture to maintain eye gaze with book); (e) Distractibility: From a rating of 1 (Child is fidgeting, rocking, or moving during the reading session; easily distracted by other events in the classroom) to a rating of 4 (If there are major distractions in the book reading, the child is minimally distracted and easily transitions back to the story reading session); (f) Verbal communication: From a rating of 1 (The child does not communicate verbally in response to storybook reading) to a rating of 4 (The child may volunteer relevant information and thoughts during the book reading); (g) Response to adult support: From a rating of 1 (Adults may provide support but, even with support, the child is minimally engaged throughout the book interaction) to a rating of 4 (Minimal/no adult support is required).

Inter-rater reliability was calculated on a randomly selected 20% of the sample, with independent coding performed by the two trained raters. Results indicated good to excellent inter-rater reliability across all COB variables, with intraclass correlation coefficients demonstrating good to excellent agreement (ICC = 0.82–0.91), depending on the specific dimension assessed (Overall impression, persistence, posture, eye gaze, distractibility, verbal communication, and response to adult support).

### 2.3 Procedure

The study was conducted in accordance with the ethical standards of the Declaration of Helsinki. Ethical approval was obtained from the Ethics Committee of the Catholic University of Valencia San Vicente Mártir (approval code: UCV/2017-2018/46). Written informed consent was obtained from all parents or legal guardians prior to participation. In addition, verbal assent was obtained from the children, and their willingness to participate was monitored throughout the assessment sessions.

The assessment of narrative ability took place in a quiet room either at the school or at the specialized intervention center attended by the children. The sessions followed the same structured sequence, with the presentation modality counterbalanced across participants: (i) The story was read by a psychologist specialized in neurodevelopmental disorders (C.F.S.) using one of two modalities: Traditional modality: printed book with illustrations and Digital modality with augmented reality (AR application on a tablet).

(ii) The story was then retold by the child, using only the images from the book as visual support (traditional modality) or the three-dimensional images in the case of augmented reality (digital modality).

Children were given the following instructions: *“I am going to tell you a story about a boy named Ismael. Listen carefully because when I finish, you will tell it to (speech language pathologist/resource teacher), who does not know it. Are you ready?”*

As indicated by the previous instruction, after viewing the story, the child was asked to retell it to a different third person—a support teacher not involved in the intervention—to ensure that there was no shared prior knowledge. During the narration, the therapist attempted to maintain the interaction as if it were a conversational exchange, avoiding presenting the task as an “interrogation.” This was achieved using phatic markers (e.g., mm, really? oh!, is that so?) and nonverbal cues (eye contact, head nodding), to indicate attentive listening. In general, the interlocutor did not intervene unless the child stopped the flow of the narrative. In such cases, continuation was prompted through immediate repetition of the child’s last utterance or through questions aimed at eliciting narrative progression (e.g., *What happened next?*, *And then what happened?*, *What else?*).

The study employed a within-subject design in which all 15 participants completed both experimental conditions: the augmented reality (AR) condition and the print-only treatment-as-usual (TAU) condition. To control for potential order effects, the sequence of condition administration was counterbalanced across participants: eight children completed the AR condition first followed by the TAU condition, whereas seven children completed the TAU condition first followed by the AR condition. Both stories were administered across the two conditions, and the assignment of story to condition was counterbalanced to minimize potential sequence and story-specific effects.

### 2.4 Data analysis

All statistical analyses were conducted using JASP (version 0.96). Prior to hypothesis testing, the assumption of normality was evaluated for each pairwise difference score using the Shapiro–Wilk test. Inspection of the results indicated that the majority of variables did not show substantial deviations from normality (i.e., *p* > 0.05), supporting the use of parametric analyses. Given the robustness of the paired-samples *t*-test to minor violations of normality, particularly in small samples, all variables were retained for parametric testing. To assess differences between the AR condition and the printed-book condition (TAU condition), a series of paired-samples *t*-tests was performed. This approach is appropriate for within-subject comparisons where the same participants are assessed under both conditions. Effect sizes were calculated using Cohen’s *d* for dependent samples, and 95% confidence intervals (CIs) were reported to provide an estimate of precision. Effect sizes were interpreted according to the conventional benchmarks proposed by Cohen (1988), where *d* = 0.20 indicates a small effect, *d* = 0.50 a medium effect, and *d* = 0.80 a large effect. Statistical significance was set at *p* < 0.05. Moreover, a sensitivity power analysis was conducted using G*Power to determine the minimum detectable effect size for the available sample. For a two-tailed paired-samples *t* test with *N* = 15, *α* = 0.05, and a statistical power of 0.80, the analysis yielded a minimum detectable effect size of Cohen’s (*d* = 0.78), indicating that the study was adequately powered to detect large within-subject effects.

## 3 Results

The primary objective of the present analysis was to compare participants’ performance under the AR condition versus the only printed-book condition across multiple narrative, socio-cognitive, and reading-oriented outcomes, using a within-subject design. Descriptive statistics indicated consistently higher mean scores in the AR condition across most variables, suggesting a general trend toward improved performance. Inferential analyses were conducted using paired-samples *t*-tests, with the Holm–Bonferroni correction applied to control for multiple comparisons (Table 2).

**Table 2:** Paired samples *t*-tests comparing AR and TAU conditions in ASD children (*n* = 15).

Variable	AR condition	TAU condition	*t*	df	*p*	Cohen’s *d*	95% CI
Number of utterances	24.67 ± 2.97	20.53 ± 6.51	2.31	14	0.036	0.59	[0.04, 1.14]
Mean Length of Utterance (MLU)	5.42 ± 1.39	4.86 ± 1.10	1.42	14	0.177	0.36	[−0.16, 0.88]
Subordination index	0.15 ± 0.14	0.09 ± 0.10	1.60	14	0.131	0.41	[−0.12, 0.94]
Number of different words	81.60 ± 38.47	58.00 ± 17.2	2.24	14	0.042	0.57	[0.02, 1.12]
Type–token ratio	0.61 ± 0.08	0.57 ± 0.06	2.29	14	0.038	0.59	[0.03, 1.13]
Content	13.30 ± 2.71	11.90 ± 2.79	1.45	14	0.169	0.37	[−0.16, 0.89]
Theory of Mind (ToM)	4.27 ± 1.62	2.13 ± 1.89	3.75	14	0.002^*^	0.97	[0.34, 1.58]
Total comprehension	17.13 ± 2.13	15.27 ± 3.39	1.82	14	0.089	0.47	[−0.07, 1.00]
COB	29.00 ± 2.93	23.40 ± 8.20	3.34	14	0.005^*^	0.86	[0.26, 1.45]

COB, Children’s Orientation to Book Reading (COB) Rating Scale (Kaderavek et al., 2014); AR, Augmented Reality; TAU, Treatment As Usual; ASD, Autism Spectrum Disorder. Bonferroni-adjusted ^*^p < 0.0056.

At the uncorrected level, statistically significant improvements were observed in several indices of narrative microstructural production. Specifically, participants produced a greater number of utterances in the AR condition compared to the printed-book condition, *t*(14) = 2.32, *p* = 0.036, with a medium effect size (*d* = 0.60). Similarly, lexical diversity, as measured by the number of different words, was significantly higher in the AR condition, *t*(14) = 2.24, *p* = 0.042, *d* = 0.58. The type–token ratio also showed a significant increase, *t*(14) = 2.29, *p* = 0.038, *d* = 0.59, indicating enhanced lexical variability. However, after applying the Holm–Bonferroni correction, these effects did not remain statistically significant (Bonferroni-adjusted *p* < 0.0056), suggesting that although the AR condition may positively influence language productivity and lexical diversity, these effects should be interpreted with caution.

No significant differences were observed for mean length of utterance (MLU), *t*(14) = 1.42, *p* = 0.177, or the subordination index, *t*(14) = 1.61, *p* = 0.131, indicating that structural aspects of language complexity were not significantly impacted by the AR condition. Regarding narrative macrostructure measures, the main ideas score did not differ significantly between conditions, *t*(14) = 1.45, *p* = 0.169. The total comprehension score also did not reveal significant differences between conditions, *t*(14) = 1.83, *p* = 0.089, although the latter approached significance and showed a moderate effect size (*d* = 0.47), suggesting a potential trend toward improvement.

In contrast, robust and statistically significant differences were observed in the use of mental state terms and global reading-orientation behaviors. Participants produced significantly more mental state terms in the AR condition, *t*(14) = 3.76, *p* = 0.002, which remained significant after Holm–Bonferroni correction (Bonferroni-adjusted *p* < 0.0056; Figure 3). The associated effect size was large (*d* = 0.97), indicating a substantial improvement in this variable. Similarly, the COB revealed significantly higher scores in the AR condition, *t*(14) = 3.35, *p* = 0.005, which also remained significant after correction (Bonferroni-adjusted *p* < 0.0056), with a large effect size (*d* = 0.86; Figure 4). This finding suggests that the AR condition produced meaningful improvements in overall reading-orientation behaviors.

**Figure 3:**
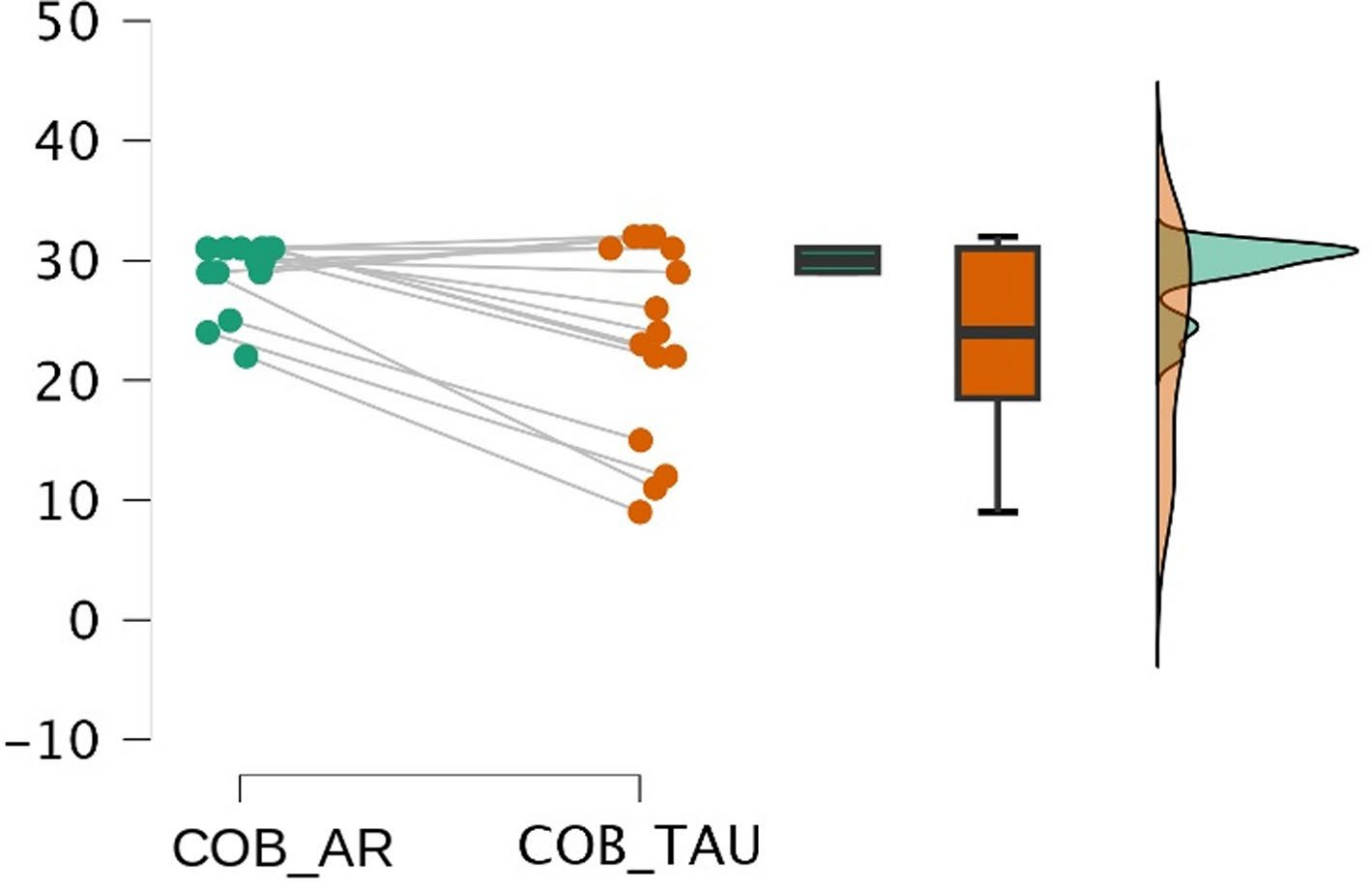
Raincloud plot of the difference between ToM in AR (augmented reality) condition and ToM in TAU condition in children with ASD.

**Figure 4:**
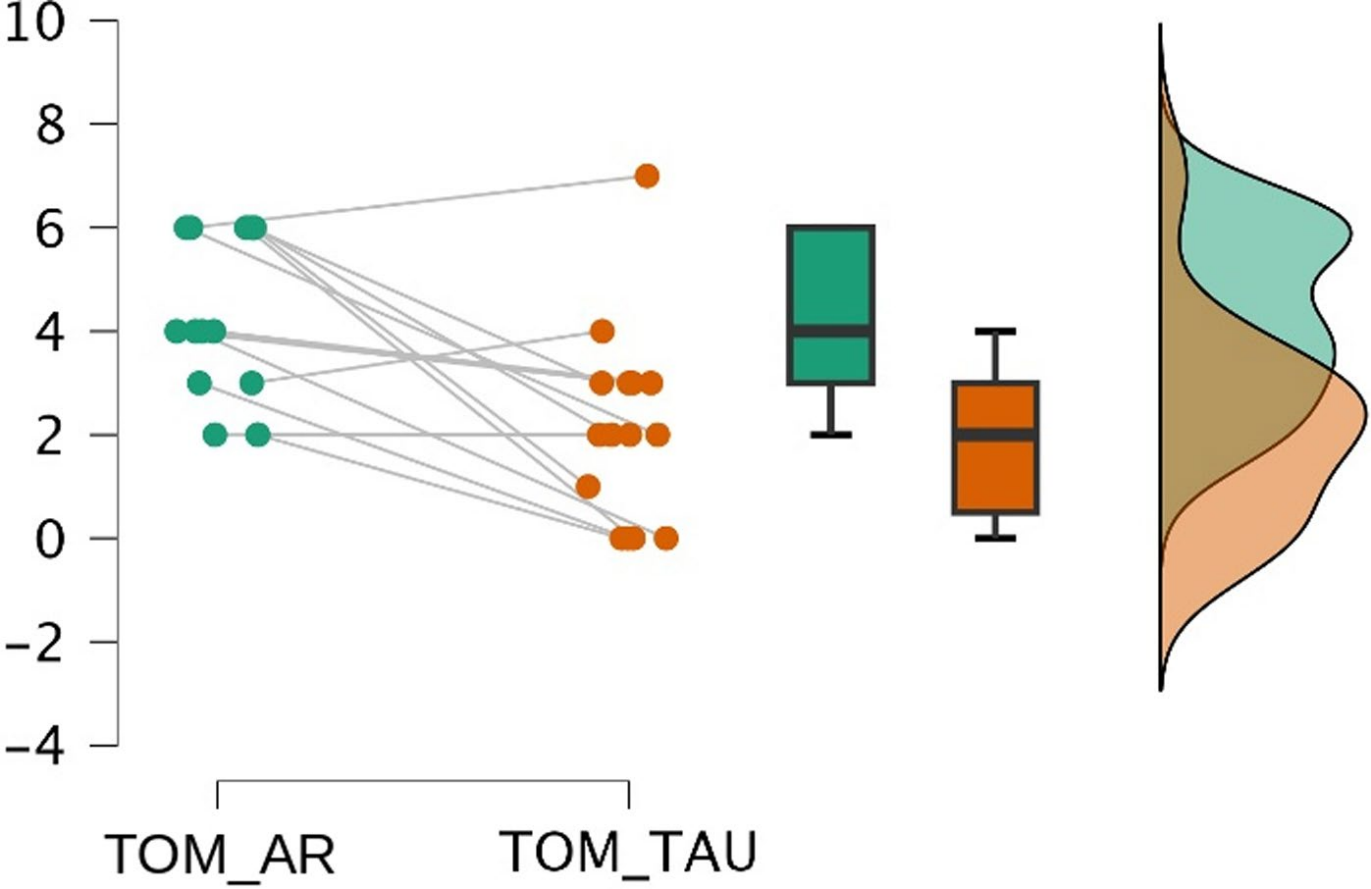
Raincloud plot of the difference between COB in AR (augmented reality) condition and COB in TAU condition in children with ASD.

Taken together, the results indicate a differential pattern of effects across domains. After controlling for multiple comparisons, the AR condition demonstrated robust and reliable effects primarily in socio-cognitive indicators, such as mental state terms, and reading-orientation outcomes, both showing large effect sizes. In contrast, improvements in narrative microstructure measures, although initially significant and of moderate magnitude, did not withstand correction.

## 4 Discussion

The primary objective of the present study was to examine the impact of augmented reality on narrative production, comprehension, sociocognitive indicators and book-reading-oriented behaviors in children with Autism Spectrum Disorder (ASD). Through a within-subject design, an AR-enriched digital modality was compared to the traditional paper-based condition. The findings reveal a differential pattern of benefits, with significant impacts on socio-cognitive indicators and behavioral engagement. The first specific objective of this study was to determine whether the AR condition would lead to higher levels of reading-oriented behavior compared to the traditional paper mode. As hypothesized, the AR condition resulted in significantly higher scores on the Children’s Orientation to Book Reading (COB) scale. The statistically significant difference and the large effect size indicate that the AR modality substantially improved overall engagement, persistence, and attentional focus. These results align with previous research suggesting that digital and multimodal formats can enhance the motivation and involvement of children with ASD by providing a more stimulating and interactive literacy experience (Richter and Courage, 2017; Chen et al., 2015; Diehm et al., 2020). In fact, digital tools have been shown to act as intrinsic reinforcers that increase enthusiasm in cognitively demanding tasks, such as shared reading. In this regard, Richter and Courage (2017) had already observed that children show significantly greater engagement and higher levels of persistence when interacting with electronic formats compared to printed ones.

The second objective focused on examining the differential effects of AR on narrative production, both at the microstructural and macrostructural levels. It was expected that the enhanced visual support of AR would facilitate richer linguistic production. However, although the initial uncorrected analyses indicated significant improvements in microstructural language parameters—such as the number of utterances, number of different words, and type-token ratio—these effects were no longer significant after applying the Holm–Bonferroni correction. Furthermore, structural measures such as Mean Length of Utterance (MLU) and the subordination index showed no significant differences. This suggests that while AR may serve as a prompt for increased verbal output, it does not fundamentally alter the underlying syntactic complexity of the children’s language. Regarding narrative macrostructure, the results indicated that the ability to convey the main ideas of the story did not differ significantly between the AR condition and the traditional book. This finding suggests that global discourse structure and the prioritization of key information are high-level cognitive processes that exhibit greater stability and resistance to immediate changes derived solely from the presentation medium. Similarly, the total comprehension score did not reveal statistically significant differences between the two conditions. Nevertheless, it is essential to highlight that this result showed a trend toward significance with a moderate effect size in favor of the AR condition. This “trend effect” suggests that while a single exposure session may not be sufficient to drastically alter comprehension levels, the dynamic visual stimulus provided by AR might be facilitating more fluid access to the plot. This positive tendency partially aligns with the findings of Wainwright et al. (2020), who indicate that the value of technology in ASD lies not only in its visual appeal but in its capacity to direct attention toward the critical elements of the story. The fact that comprehension approached significance could indicate that AR might help children connect sequential events that, in a paper format, require greater inferential effort. Moreover, the absence of full significance in comprehension coincides with previous findings suggesting a gap between visual attention and deep information processing (Wainwright et al., 2020). In other words, the absence of statistically robust effects might be due to the “visual attention-to-processing gap”: children may be highly engaged with the 3D images without necessarily translating that visual interest into deep semantic recall. Nonetheless, the observed effect size allows for the hypothesis that, with a larger sample size, the visual scaffolding of AR could become an effective tool for improving oral comprehension. This would be achieved by reducing the ambiguity of static illustrations and providing a more explicit representation of the narrative’s causal relationships. The observed trend suggests that with repeated exposure, the visual scaffolding of AR could lead to better story recall by reducing the inferential load required to connect sequential events. Future studies with larger samples and longitudinal designs are needed to confirm whether these effects strengthen over time and reach statistical significance.

The final objective of the current study was to analyze whether the AR condition promoted a greater use of socio-cognitive references, such as terms related to mental states. As predicted, participants produced significantly more mental state terms in the AR condition. This finding remained significant even after controlling for multiple comparisons and yielded a large effect size. Therefore, these results indicate that the three-dimensional and dynamic nature of AR images may provide essential scaffolding that helps children with ASD interpret and verbalize characters’ intentions, emotions, and beliefs, which are often difficult to grasp from static two-dimensional illustrations. Given that children with ASD process information more efficiently when it is presented in a structured manner, it is possible that the ability of AR to transform static images into predictable dynamic sequences may have reduced the cognitive load. By removing the mental effort required to imagine movement or transitions between scenes, the child can focus their cognitive resources on the narrative content. This observation aligns with Diehm et al. (2020), who argue that dynamic visual supports, such as animation, facilitate access to crucial information regarding actions and event sequencing. Furthermore, this dynamic visual support allows for the explication of socio-cognitive elements that are typically implicit and difficult for this population to decode. AR animation can more clearly represent characters’ facial expressions and intentions—core elements of Theory of Mind (ToM). As Wainwright et al. (2020) point out, the clinical value of this technology lies in its capacity to direct attention toward key plot elements, enabling a deeper interpretation of the story. The robustness of the effect on socio-cognitive indicators (Theory of Mind) is perhaps the most relevant finding of the present study, as these abilities are typically impaired in children with ASD and are closely linked to their narrative production (Losh and Capps, 2003; Peristeri et al., 2017).

From a neurocognitive perspective, the benefits observed in the AR condition may be closely linked to the strong visual processing profiles often characteristic of individuals with ASD. According to the Weak Central Coherence theory, children with autism frequently demonstrate a bias toward local, detail-oriented information at the expense of global integration. In traditional paper-based reading, the demand to integrate static, fragmented illustrations into a coherent narrative whole may overwhelm their integrative processing capacity. However, AR’s ability to present information through simultaneous and explicit multimodal cues—where movement, 3D depth, and spatial consistency are provided in real-time—may mitigate these integration difficulties. By offering a more visually cohesive stimulus, AR likely facilitates the transition from “detail-focused” perception to a more “global” understanding of the narrative context, thereby allowing the child to allocate more cognitive resources to the social and causal dimensions of the story.

In summary, the results of the current study suggest a differential impact of AR depending on the narrative domain evaluated. While basic linguistic structures remained stable, AR significantly boosted the socio-cognitive richness of the narratives and the children’s behavioral engagement. The preference for visual and structured input in children with ASD indicates that AR may act as a “cognitive filter.” By highlighting salient cues and guiding attention toward relevant events, AR may reduce the “noise” often found in complex social scenes. This supports the idea that digital formats are not just more “fun,” but more “legible,” allowing for sustained focus that traditional paper sometimes fails to provide. In this way, AR could serve as a bridge to overcome the gap in Theory of Mind (ToM) by providing a more transparent representation of social causality.

### 4.1 Clinical and educational implications

The findings of the present study underscore the potential of AR as a scaffolding tool for clinical and educational professionals. AR implementation has several relevant implications for both clinical practice and educational settings working with children with Autism Spectrum Disorder (ASD). First, the robust effects observed in reading-oriented behaviors suggest that AR constitutes a valuable tool for optimizing assessment conditions. By significantly increasing engagement, persistence, and attentional focus during shared reading tasks, AR may help minimize common barriers frequently encountered in clinical evaluations with this population, such as distractibility, low motivation, and reduced task involvement. From an evaluative perspective, this increased orientation to the task may allow professionals to obtain a more accurate representation of the child’s narrative and socio-cognitive abilities, reducing the risk of underestimating performance due to non-linguistic factors.

Second, the significant improvement in the use of mental state terms has direct implications for interventions targeting social cognition and Theory of Mind (ToM). Narrative contexts are widely recognized as a privileged framework for fostering socio-cognitive development, as they require children to infer and articulate characters’ intentions, emotions, and beliefs. The present results suggest that AR-enhanced narratives can function as an effective scaffold to support this process in children with ASD by making otherwise implicit socio-cognitive cues more explicit and visually accessible. Clinically, this points to the potential of AR-based materials as complementary resources in interventions aimed at improving pragmatic language skills, perspective-taking, and emotional understanding.

From an educational standpoint, the observed increase in engagement and socio-cognitive references indicates that AR may be especially useful in inclusive classroom settings, where maintaining sustained attention and active participation in literacy activities can be challenging for students with ASD. Integrating AR into shared reading or storytelling activities may promote more meaningful participation and facilitate access to narrative content without fundamentally altering curricular objectives. Importantly, the results suggest that AR does not replace traditional literacy practices but rather enhances them by enriching the narrative context and supporting comprehension and social interpretation.

Furthermore, the absence of strong effects on narrative macrostructure and syntactic complexity highlights an important consideration for both clinicians and educators: AR should be conceptualized as a supportive tool rather than a standalone intervention for language development. While the technology appears particularly effective in enhancing engagement and socio-cognitive expression, improvements in higher-level discourse organization and linguistic structure may require explicit instructional strategies and repeated intervention over time. Practitioners should therefore integrate AR within comprehensive, goal-oriented intervention programs, combining it with evidence-based approaches such as dialogic reading, explicit narrative instruction, and guided metalinguistic reflection.

Finally, the results underscore the importance of aligning assessment and intervention tools with the technological environments that increasingly characterize children’s everyday experiences. Incorporating AR into clinical and educational practice may enhance ecological validity and increase motivation, allowing children with ASD to demonstrate their abilities under conditions that are both supportive and developmentally relevant. Nevertheless, careful selection and structured use of AR resources are essential to ensure that the technology directs attention toward key narrative elements rather than acting as a source of distraction. When used judiciously, AR has the potential to enrich narrative-based assessment and intervention, particularly by fostering engagement and supporting socio-cognitive processing in children with ASD.

### 4.2 Limitations and future research

This study has several limitations that should be acknowledged. First, the small sample size limits the generalizability of the findings and reduces the statistical power to detect subtle changes in linguistic microstructure after corrections for multiple comparisons. Therefore, future research should examine whether prolonged exposure to AR can consolidate improvements in narrative ability. Finally, the variability in the results suggests that future research should focus on individual profiles. Factors such as sensory sensitivity or baseline IQ might determine who benefits most from AR. For instance, it would be relevant to explore whether each child’s sensory profile (e.g., visual or auditory sensitivity) modulates the effectiveness of tablet-based interventions. Further research could also compare the effects of AR not only with printed books but also with standard digital formats (non-augmented e-books), in order to isolate the specific contribution of three-dimensionality.

Furthermore, the promising advantages of AR should be interpreted in light of current limitations in its application in pediatric and educational contexts. A survey by Wang (2022) among families of preschool children showed that a considerable proportion of parents are not familiar with AR technology, and that many preschool children are not able to use mobile devices independently, requiring adult assistance. In addition, a significant number of parents reported that existing AR picture books lack sufficiently engaging interaction. Therefore, the effectiveness of AR in early childhood contexts depends not only on the technology itself but also on its pedagogical and design quality, and these limitations must be carefully considered when implementing AR-based tools.

This study provides preliminary evidence that AR-enhanced narrative elicitation may improve socio-cognitive language use and reading-oriented behaviors in children with ASD without intellectual disability. However, AR did not produce robust effects on narrative macrostructure, syntactic complexity, or comprehension after correction for multiple comparisons. These findings suggest that AR may be particularly useful as a scaffold for engagement and mental state language, while broader narrative improvements may require repeated exposure and explicit intervention.

An additional consideration concerns the potential novelty effect associated with the AR condition. Because AR represented a more technologically enriched and visually dynamic format than the conventional printed-book condition, part of the observed increase in engagement and reading-oriented behavior may reflect participants’ initial interest in, or attraction to, the technology itself rather than an effect specific to the augmented features. The present design does not allow these influences to be fully disentangled. Future studies should therefore include repeated exposure to AR to determine whether the observed benefits persist once the technology becomes familiar, as well as an additional non-augmented digital condition to distinguish the specific contribution of AR from the more general effects of digital presentation, interactivity, and technological novelty.

## 5 Conclusion

The present study provides preliminary evidence that AR-enhanced narrative activities may offer meaningful benefits for children with ASD, particularly in relation to reading-oriented engagement and socio-cognitive language use. Participants demonstrated greater behavioral engagement and increased use of mental-state references in the AR condition, whereas no robust improvements were observed in narrative macrostructure, syntactic complexity, or overall comprehension. These findings suggest that AR may be particularly valuable as a visual and interactive scaffold for directing attention toward socially and narratively relevant information rather than as a general mechanism for improving all dimensions of narrative performance. Nevertheless, the findings should be interpreted cautiously given the small sample size and the possibility that technological novelty contributed to the increased engagement observed in the AR condition. Further research using larger samples, repeated AR exposure, longitudinal designs, and non-augmented digital comparison conditions is needed to determine whether these benefits are sustained over time and can be attributed specifically to AR rather than to the novelty or general interactivity of digital technology. Overall, AR should be viewed as a promising complementary resource for narrative-based educational and clinical activities in children with ASD, rather than as a replacement for established literacy and language-intervention practices.

## Data Availability

The raw data supporting the conclusions of this article will be made available by the authors, without undue reservation.

## References

[ref1] Adornetti I. Chiera A. Altavilla D. Deriu V. Marini A. Gobbo M. . (2024). Defining the characteristics of story production of autistic children: a multilevel analysis. J. Autism Dev. Disord. 54, 3759–3776. doi: 10.1007/s10803-023-06096-2, 37653117 PMC11461702

[ref2] Aguado G. (1995). El desarrollo del lenguaje de 0 a 3 años: Bases para un diseño curricular en la Educación Infantil. Madrid: Cepe.

[ref3] American Psychological Association (2020). Publication Manual of the American Psychological Association. 7th Edn. Washington, DC: American Psychological Association.

[ref4] Astington J. W. Baird J. A. eds. (2005). Why Language Matters for Theory of Mind. Oxford: Oxford University Press.

[ref5] Baixauli I. Colomer C. Roselló B. Miranda A. (2016). Narratives of children with high-functioning autism spectrum disorder: a meta-analysis. Res. Dev. Disabil. 59, 234–254. doi: 10.1016/j.ridd.2016.09.007, 27643571

[ref6] Baixauli-Fortea I. Gómez-García S. Andrés-Sebastiá M. d. P. Berenguer-Forner C. (2019). Una propuesta de intervención para niños con trastorno del espectro autista a través de la lectura dialógica y la realidad aumentada. Edetania: Estudios y Propuestas Socio-educativas 56, 135–150.

[ref7] Berenguer C. Baixauli I. Gómez S. Andrés M. E. P. De Stasio S. (2020). Exploring the impact of augmented reality in children and adolescents with autism spectrum disorder: a systematic review. Int. J. Environ. Res. Public Health 17:6143. doi: 10.3390/ijerph17176143 PMC7504463 32847074

[ref8] Bishop D. V. M. (2004). Expression, Reception and Recall of Narrative Instrument (ERRNI). London: Pearson Assessment.

[ref9] Brown R. (1973). A First Language: The Early Stages. Cambridge, Massachusets: Harvard University Press.

[ref10] Chen C. H. Lee I. J. Lin L. Y. (2015). Augmented reality-based self-learning system to improve the literacy abilities of children with autism spectrum disorder. Res. Autism Spectr. Disord. 9, 21–25. doi: 10.1016/j.rasd.2014.10.004, 42574925

[ref11] Chen C.-H. Lee I.-J. Lin L.-Y. (2016). Augmented reality-based video-modeling storybook of nonverbal facial cues for children with autism spectrum disorder to improve their perceptions and judgments of facial expressions and emotions. Comput. Hum. Behav. 55, 477–485. doi: 10.1016/j.chb.2015.09.033

[ref1101] Cohen J. (1988). Statistical power analysis for the behavioral sciences (2nd ed.). Hillsdale, NJ: Erlbaum.

[ref14] Diehl J. J. Bennetto L. Young E. C. (2006). Story recall and narrative coherence of high-functioning children with autism spectrum disorders. J. Abnor. Child Psychol. 34, 87–102. doi: 10.1007/s10802-005-9003-x, 16485176

[ref15] Diehm E. Wood C. Puhlman J. Callendar M. (2020). Young children’s narrative retell in response to static and animated stories. Int. J. Lan. Commun. Disord. 55, 359–372. doi: 10.1111/1460-6984.12523, 31925888 PMC9292601

[ref16] Domingo L. Baixauli I. Berenguer C. (2026). “Buenas noches, gorila”: Evaluación de la narración en niños con trastornos del espectro autista. Medicina 86, 58–64. 41696841

[ref17] Duinmeijer I. de Jong J. Scheper A. (2012). Narrative abilities, memory and attention in children with a specific language impairment. Int. J. Lan. Commun. Disord. 47, 542–555. doi: 10.1111/j.1460-6984.2012.00164.x, 22938065

[ref18] Escobedo L. Tentori M. Quintana E. Favela J. García-Rosas D. (2014). Using augmented reality to help children with autism stay focused. IEEE Pervasive Comput. 13, 38–46. doi: 10.1109/MPRV.2014.19

[ref19] Ferretti F. Adornetti I. Chiera A. Nicchiarelli S. Valeri G. Magni R. . (2018). Time and narrative: an investigation of storytelling abilities in children with autism spectrum disorder. Front. Psychol. 9:944. doi: 10.3389/fpsyg.2018.00944, 29971024 PMC6018079

[ref20] Fuentes C. Gómez S. De Stasio S. Berenguer C. (2025). Augmented reality and learning-cognitive outcomes in autism spectrum disorder: a systematic review. Children (Basel, Switzerland) 12:493. doi: 10.3390/children12040493, 40310103 PMC12026450

[ref21] Gazella J. Stockman I. J. (2003). Children’s story retelling under different modality and task conditions: implications for standardizing language sampling procedures. Am. J. Speech Lang. Pathol. 12, 61–72. doi: 10.1044/1058-0360(2003/053), 12680814

[ref22] Gómez Soledad (2019). Ejemplo libro El cumpleaños de Ismael: Colección las historias de Ismael [Video]. YouTube. Available online at: https://youtu.be/aVDtQ1ms_zY (Accessed August 7, 2026).

[ref23] Gutiérrez-Clellen V. F. Restrepo M. Bedore L. Peña E. Anderson R. (2000). Language sample analysis in Spanish-speaking children: methodological considerations. Lang. Speech Hear. Serv. Sch. 31, 88–98. doi: 10.1044/0161-1461.3101.88, 27764338

[ref24] Happé F. Frith U. (2006). The weak coherence account: detail-focused cognitive style in autism spectrum disorders. J. Autism Dev. Disord. 36, 5–25. doi: 10.1007/s10803-005-0039-0, 16450045

[ref25] He C. Miyamoto S. Lin Y. (2025). Narratives of Chinese kindergarteners with autism spectrum disorder: comparison with typically developing children. Clin. Linguist. Phon. 39, 1117–1141. doi: 10.1080/02699206.2025.2451968, 39853168

[ref26] Hewitt L. E. (2019). Narrative as a critical context for advanced language development in autism spectrum disorder. Perspect. ASHA Spec. Interest Groups 4, 430–437. doi: 10.1044/2019_PERS-SIG1-2018-0021

[ref27] Hughes D. L. McGillivray L. A. Schmidek M. (1997). Guide to Narrative Language: Procedures for Assessment. Eau Claire: Wisconsin, Thinking Publications.

[ref28] Hunt K.W. (1965). Grammatical Structures Written at Three Grade Levels. National Council of Teachers of English Research Report No. 3. National Council of Teachers of English

[ref30] Kaderavek J. Guo Y. Justice L. (2014). Validity of the children’s orientation to book reading rating scale. J. Res. Read. 37, 159–178. doi: 10.1111/j.1467-9817.2012.01528.x, 40046247

[ref31] Kaufman A. S. Kaufman N. L. (2004). *KBIT-2: Test Breve de Inteligencia de Kaufman [Kaufman Brief Intelligence Test-Second Edition]* (Madrid: Traducción de TEA Ediciones). TEA Ediciones.

[ref32] Kazemi Y. Nakhshab M. Arabpour M. (2014). Narrative assessment methods in children: a systematic review. J. Res. Rehabil. Sci. 10:704–719. doi: 10.22122/jrrs.v10i5.2090

[ref33] Kljun M. Geroimenko V. Pucihar K. (2020). “Augmented reality in education,” in Augmented Reality in Education, ed. Geroimenko V. (London: Springer), 3–21.

[ref34] Klop D. Engelbrecht L. (2013). The effect of two different visual presentation modalities on the narratives of mainstream grade 3 children. S. Afr. J. Commun. Disord. 60, 21–26. doi: 10.7196/sajcd.242 25158369

[ref35] Kucirkova N. (2019). Children’s reading with digital books: past moving quickly to the future. Child Dev. Perspect. 13, 208–214. doi: 10.1111/cdep.12339

[ref36] Lauricella A. R. Barr R. Calvert S. L. (2014). Parent–child interactions during traditional and computer storybook reading for children’s comprehension: implications for electronic storybook design. Int. J. Child-Comput. Interact. 2, 17–25. doi: 10.1016/j.ijcci.2014.07.001, 42574925

[ref37] Leung W. S. Blauw F. F. (2020). “An augmented reality approach to delivering a connected digital forensics training experience,” in Information Science and Applications, ICISA 2019, eds. Kim K. J. Kim H.-Y., Lecture Notes in Electrical Engineering, vol. 621 (London: Springer), 353–361.

[ref39] Losh M. Capps L. (2003). Narrative ability in high-functioning children with autism or Asperger’s syndrome. J. Child Psychol. Psychiatry 44, 242–254. doi: 10.1023/A:1024446215446, 12908827

[ref41] Miller J. F. Iglesias A. (2012). *Systematic Analysis of Language Transcripts (SALT)* (Versión 12) [Software de computación]. Middleton: Wisconsin: SALT Software, LLC

[ref42] Norbury C. F. Bishop D. V. (2003). Narrative skills of children with communication impairments. Int. J. Lang. Commun. Disord. 38, 287–313. doi: 10.1080/136820310000108133, 12851080

[ref43] Norbury C. F. Gemmell T. Paul R. (2014). Pragmatics abilities in narrative production: a cross-disorder comparison. J. Child Lang. 41, 485–510. doi: 10.1017/S030500091300007X, 23632039

[ref44] O’Byrne W. I. Houser K. Stone R. White M. (2018). Digital storytelling in early childhood: student illustrations shaping social interactions. Front. Psychol. 9:1800. doi: 10.3389/fpsyg.2018.01800, 30364158 PMC6191536

[ref45] O’Neill D. K. Pearce M. J. Pick J. L. (2004). Preschool children’s narratives and performance on the Peabody individualized achievement test–revised: evidence of a relation between early narrative and later mathematical ability. First Lang. 24, 149–183. doi: 10.1177/0142723704043529

[ref46] Peristeri E. Andreou M. Tsimpli I. M. (2017). Syntactic and story structure complexity in the narratives of high- and low-language ability children with autism spectrum disorder. Front. Psychol. 8:2017. doi: 10.3389/fpsyg.2017.02027, 29209258 PMC5701940

[ref47] Peristeri E. Drakoulaki K. Boznou A. Nerantzini M. Varlokosta S. (2025). Linguistic Markers of Autism Spectrum Disorder in Narrative Production: Evidence From the Monkey Cartoon Storytelling Task of the Autism Diagnostic Observation Schedule. Autism Dev. lang. Impair, 10:23969415251331045. doi: 10.1177/23969415251331045 PMC12078983 40376279

[ref48] Premack D. Woodruff G. (1978). Does the chimpanzee have a Theory of Mind? Behav. Brain Sci. 1, 515–526. doi: 10.1017/S0140525X00076512

[ref49] Renfrew C. (2010). Bus Story Test: Revised Edition (The Renfrew Language Scales). London: Routledge.

[ref50] Richards B. (1987). Type/token ratios: what do they really tell us? J. Child Lan. 14, 201–209. doi: 10.1017/S0305000900012885, 3611238

[ref51] Richter A. Courage M. (2017). Comparing electronic and paper storybooks for preschoolers: attention, engagement, and recall. J. Appl. Dev. Psychol. 48, 92–102. doi: 10.1016/j.appdev.2017.01.002

[ref53] Schneider P. Dubé R. V. (1997). Effect of pictorial versus oral story presentation on children's use of referring expressions in retell. First Lan. 17, 283–302. doi: 10.1177/014272379701705113

[ref54] Şimşek E. E. (2024). The effect of augmented reality storybooks on the story comprehension and retelling of preschool children. Front. Psychol. 15:1459264. doi: 10.3389/fpsyg.2024.1459264, 39439758 PMC11493726

[ref55] Tager-Flusberg H. Rogers S. Cooper J. Landa R. Lord C. Paul R. . (2009). Defining spoken language benchmarks and selecting measures of expressive language development for young children with autism spectrum disorders. J. Speech Lang. Hear. Res.: JSLHR 52, 643–652. doi: 10.1044/1092-4388(2009/08-0136), 19380608 PMC2819321

[ref56] Wainwright B. R. Allen M. L. Cain K. (2020). Narrative comprehension and engagement with e-books vs. paper-books in autism spectrum condition. Autism Dev. lang. Impair, 5:2396941520917943. doi: 10.1177/2396941520917943 PMC9620454 36381546

[ref57] Wang R. (2022). Application of augmented reality technology in Children’s picture books based on educational psychology. Front. Psychol. 13:782958. doi: 10.3389/fpsyg.2022.782958, 35185721 PMC8850626

